# Gender specific factors associated with having stopped smoking among in-school adolescents in Ukraine: results from the Global Youth Tobacco Survey 2005

**DOI:** 10.1186/1756-0500-3-76

**Published:** 2010-03-16

**Authors:** Alice Hazemba, Seter Siziya, Adamson S Muula, Emmanuel Rudatsikira

**Affiliations:** 1 Department of Community Medicine, University of Zambia Medical School, Lusaka, Zambia; 2 Department of Community Health, University of Malawi, Blantyre, Malawi; 3 Departments of Global Health, Biostatistics and Epidemiology, School of Public Health, Loma Linda University, California, USA

## Abstract

**Background:**

The prevalence of cigarette smoking in Ukraine is different between genders and is among the highest in the world. There is need to identify gender-specific factors that are associated with having stopped smoking among adolescents.

**Findings:**

We used data from the Ukraine Global Youth Tobacco Survey 2005. We carried out a backward stepwise logistic regression analysis with having stopped smoking as the outcome.

Altogether, 2800 adolescents reported having ever smoked cigarettes. Overall 64.1% (63.4% male, and 65.5% female) adolescents reported having stopped smoking. Male adolescents who stated that smoking decreases body weight were 25% more likely, while female adolescents were 9% less likely to stop smoking. While male adolescents who received support on how to stop smoking from a family member were 7% less likely, female adolescents were 60% more likely to stop smoking. Furthermore, while male adolescents who received a lecture on the harmful effects of smoking were 10% less likely, female adolescents were 9% more likely to stop smoking. Finally both male and female adolescents who were sure or most probably that they would not smoke a cigarette offered to them by their best friends were more likely, and those adolescents who were sure that smoking is harmful to health were less likely to stop smoking.

**Conclusions:**

Our study has identified some factors that are associated with having quit smoking that are gender-specific. We believe public health programs targeting adolescent smoking should consider these factors in their design and implementation of gender sensitive interventions.

## Background

The prevalence of tobacco smoking in Ukraine is among the highest in the world [1,2]. Furthermore, the age of initiation is getting lower [3]. In the Kiev Global Youth Tobacco survey conducted in 1999, 41% of 13 to 15 year old in-school reported being current cigarette smokers [4,5].

The Health Belief Model (HBM) is a psychological model that aims to explain and predict health-related behaviours. The model focuses on the attitudes and beliefs of individuals [6,7]. One other aspect of the HBM is the role of cues of action such as a lesson in school or a family member who discusses the harmful effects of smoking with the adolescent. The HBM has been partly used in predicting health behaviour, including smoking, among college students [8]. We used this model to identify factors that we used in the analysis.

While the incidence and prevalence of smoking in Ukraine has been reported to be growing [2], there are limited data on the cessation of smoking among adolescents and more so on gender-specific correlates for stopping smoking. In order to inform smoking prevention programs in Ukraine, we explored the gender-specific correlates of self reported cessation of smoking among adolescents.

## Methods

### Study design and data collection

Our study involved secondary analysis of cross sectional data from the Ukraine Global School Youth Tobacco Survey (GYTS) conducted in 2005. The GYTS uses a two-stage probability sampling technique, in which schools are the primary sampling units [9]. The core GTYS questionnaire was adapted to the Ukrainian needs and does not match the core questionnaire.

### Data analysis

Our data analysis was restricted to smoking participants and those who had smoked cigarettes but were no longer smokers. Data analysis was performed using SPSS version 14.0 software. A weighting factor was used in the analysis to reflect the likelihood of sampling each student and to reduce bias by compensating for differing patterns of non response. We obtained frequencies as estimation of prevalence of the main outcome (ever having stopped smoking), and other descriptive characteristics of the sample. We report unadjusted odds ratios (OR) from bivariate analyses. We also conducted a backward stepwise logistic regression analysis to estimate the associations between relevant predictor variables and the outcome, and we report adjusted odds ratios (AOR) with their 95% confidence intervals (CI) from this analysis.

## Results

Data on whether participants who had ever smoked but had stopped smoking cigarettes or were still smokers were available from 2800 out of 7727 participants. Half (50.0%) of the participants were males, and 35.5% of the respondents (35.7% of males, and 35.4% of females) were of age 14 years. Overall, 60.6% of the participants (64.2% males and 56.3% females) were current smokers and 64.3% of the adolescents (63.4% of males, and 65.5% of females) reported having stopped smoking cigarettes. The period since they stopped smoking varied from less than 1 month to 3 years or more. Further description of the sample is shown in table 1.

**Table 1:** Description of the sample

	Total	Male	Female
	n*(%)**	n*(%)**	n*(%)**
Age (years)			
<13	948 (7.7)	448 (7.6)	500 (7.9)
13	2422 (26.5)	1130 (25.2)	1292 (27.8)
14	2516 (35.5)	1265 (35.7)	1251 (35.4)
15+	1627 (30.2)	841 (31.4)	786 (29.0)
If one of your best friends proposes you a cigarette will you smoke?			
For sure, no	3305 (44.0)	1686 (45.8)	1619 (42.3)
Most probably, no	3069 (38.0)	1341 (33.5)	1728 (42.4)
Most probably, yes	476 (8.0)	272 (8.7)	204 (7.2)
For sure, yes	623 (10.1)	367 (12.0)	256 (8.2)
How do you think, in what way does cigarette smoking affect body weight?			
Weight increases	569 (8.9)	309 (10.0)	260 (7.8)
Weight decreases	4418 (54.9)	2059 (52.0)	2359 (57.8)
Weight doesn't change	2498 (36.2)	1305 (38.0)	1193 (34.5)
How do you think, is cigarette smoking harmful for your health?			
For sure, yes	3128 (46.3)	1568 (45.9)	1560 (46.6)
Most probably, yes	3490 (43.1)	1599 (40.5)	1891 (45.6)
For sure, no	437 (5.8)	272 (7.7)	165 (3.8)
Most probably, no	408 (4.9)	226 (5.9)	182 (3.9)
Have you ever got a support or advice how to stop smoking?			
Yes, in the frame of the programme or from the professional (doctor, nurse or psychologist)	573 (17.3)	351 (18.6)	222 (15.6)
Yes, from my friend	734 (24.3)	419 (23.7)	315 (25.0)
Yes, from the member of my family	534 (18.6)	342 (21.3)	192 (15.2)
Yes, from several sources	337 (13.8)	196 (14.1)	141 (13.4)
No	661 (26.1)	334 (22.3)	327 (30.8)

* unweighted frequency

** weighted percent

### Association of age with stopping smoking

Compared to adolescents aged 15 years old and older, both male and female adolescents who were of age less than 13 or were 14 years old were less likely to stop smoking, while those who were of age 13 years were more likely to stop smoking cigarettes (Table 2).

**Table 2:** Association of age and stopping smoking

	Adjusted Odds Ratio (95% Confidence Interval)	
Factor	Males	Females
Age (years)		
<13	0.95 (0.90, 1.00)	0.79 (0.72, 0.86)
13	1.83 (1.76, 1.90)	1.97 (1.88, 2.07)
14	0.84 (0.82, 0.87)	0.76 (0.73, 0.79)
15+	1	1

The analysis adjusted for all factors in tables 3 to 5.

### Best friend as source for cigarettes

Both male and female adolescents who would definitely not accept an offer of a cigarette from a close friend to smoke it were more likely to have stopped smoking cigarettes compared to adolescents who would definitely accept an offer of a cigarette from a best friend and smoke it (AOR = 3.02 for males and AOR = 3.62 for females) as shown in table 3.

**Table 3:** Associations of source of cigarettes and stopping smoking

	Adjusted Odds Ratio (95% Confidence Interval)	
Factor	Males	Females
If one of your best friends proposes you a cigarette will you smoke?		
For sure, no	2.93 (2.85, 3.02)	3.62 (3.48, 3.76)
Most probably, no	1.95 (1.89, 2.01)	1.69 (1.64, 1.75)
Most probably, yes	0.39 (0.38, 0.40)	0.39 (0.38, 0.40)
For sure, yes	1	1

The analysis adjusted for all factors in tables 2, 4 and 5.

### Harmful effects of cigarette smoking

While male adolescents who had a lecture in the previous year of the survey on harmful effects of smoking were 10% (AOR = 0.90) less likely to have stopped smoking, female adolescents were 9% (AOR = 1.09) more likely to have stopped smoking compared to adolescents who did not have such a lecture. Male adolescents who reported that cigarette smoking decreases body weight were 25% (AOR = 1.25) more likely to stop smoking, while female adolescents were 9% (AOR = 0.90) less likely to have stopped smoking compared to adolescents who said that cigarette smoking did not affect body weight.

Both male and female adolescents who reported that cigarette smoking is definitely harmful to health were less likely to stop smoking compared to adolescents who were most probably not certain that smoking is harmful to health (AOR = 0.92 for males and AOR = 0.66 for females). Furthermore, both male and female adolescents who felt that smoking increases boy weight were more likely to stop smoking (Table 4).

**Table 4:** Associations of harmful effects of cigarette smoking with stopping smoking

	Adjusted Odds Ratio (95% Confidence Interval)	
Factor	Males	Females
How do you think, in what way does cigarette smoking affect body weight?		
Weight increases	1.11 (1.06, 1.16)	1.35 (1.28, 1.42)
Weight decreases	1.25 (1.22, 1.29)	0.91 (0.88, 0.94)
Weight doesn't change	1	1
How do you think, is cigarette smoking harmful for your health?		
For sure, yes	0.92 (0.89, 0.95)	0.66 (0.63, 0.69)
Most probably, yes	1.24 (1.20, 1.28)	0.97 (0.92, 1.01)
For sure, no	0.67 (0.63, 0.72)	1.07 (0.99, 1.17)
Most probably, no	1	1
Have you ever got a support or advice how to stop smoking?		
Did you have a lecture about the harmfulness of the smoking at one of your lessons for the last academic year?		
Yes	0.90 (0.88, 0.92)	1.09 (1.05, 1.12)
No	1	1

The analysis adjusted for all factors in tables 2 to 5.

### Sources of support or advice on how to stop smoking

Both male and female adolescents who received support or advice on how to stop smoking from a programme or professional were less likely to stop smoking (AOR = 0.91 for males, and AOR = 0.93 for females), and while male adolescents who received support or advice from a member of the family were less likely to stop smoking (AOR = 0.93), female adolescents were more likely to stop smoking (AOR = 1.60) compared to adolescents who had not ever received support or advice (Table 5).

**Table 5:** Associations of sources of support or advice on how to stop smoking with stopping smoking

	Adjusted Odds Ratio (95% Confidence Interval)	
Factor	Males	Females
Yes, in the frame of the programme or from the professional (doctor, nurse or psychologist)	0.91 (0.88, 0.95)	0.93 (0.89, 0.97)
Yes, from my friend	0.75 (0.73, 0.78)	0.96 (0.93, 0.99)
Yes, from the member of my family	0.93 (0.90, 0.96)	1.60 (1.53, 1.68)
Yes, from several sources	1.37 (1.32, 1.42)	0.83 (0.80, 0.87)
No	1	1

The analysis adjusted for all factors in tables 2 to 4.

## Discussion

Our analysis of the Ukraine Global Youth Tobacco Survey 2005 has shown that, unlike among adults [1], there was no gender difference in cigarette smoking. This finding is consistent with previous reports which have indicated that there is no gender difference in tobacco use in Europe and United States [10,11]. Using data from repeat GYTS from 100 sites around the world, Warren et al report that tobacco use among girls is likely increasing [12]. We first present a discussion of factors associated with stopping smoking that were not significantly different between gender, and then present factors associated with stopping smoking that were different between gender.

Both male and female adolescents who were younger were less likely to stop smoking suggesting that this was the age group for experimenting smoking, and those not in favour of the behaviour would have more likely stop smoking immediately. Meanwhile, adolescents who would have continued to smoke would have been less likely to stop smoking at a later age.

There were no gender differences in smoking cessation related to age, being offered a cigarette by a best friend, weight increase, and getting support or advice on how to stop smoking from a professional or friend. Both male and female adolescents who were of age less than 13 or of age 14 years, certain that cigarette smoking is harmful to health, and had received help on quitting smoking from a professional or friend were less likely to have stopped smoking than those who did not. Both male and female adolescents who were of age 13, definitely would not/most probably would not accept cigarettes from a peer, and perceived smoking increases body weight were more likely to stop smoking We believe this is important information that public health policy makers and professionals involved in the designing and delivery of interventions aimed to prevent adolescent smoking in Ukraine need to consider in designing interventions that are not gender-sensitive.

It is interesting to note that both male and female adolescents who reported that they would definitely not accept a cigarette from a peer were more likely to have stopped smoking that those who would not. One sense, this would suggest that adolescents who are committed to no longer smoke were more successful to have stopped smoking. Alternatively, our finding may suggest that adolescents who have already quit smoking are more likely not to accept a cigarette from a peer than those who are still smoking. Due to the cross sectional design of our study, however, it is not possible to tease out which of the two explanations may be more likely. It is however obvious that comparing adolescents who have stopped smoking to those who are still smoking, those that have stopped smoking reported that they would not accept a cigarette offer from a peer more than those that are still smoking. Smoking cessation programs should seriously consider the role of peers in facilitating continued smoking in adolescents who may wish to quit.

We found that both male and female adolescents who reported having received advice on how to stop smoking from professionals or friends were less likely to stop smoking suggesting that these sources may not have regarded as credible by the adolescents; the reason partly being that some professionals also smoke cigarettes.

Findings from this study indicate that there were gender differences in smoking cessation related to the perception of the effects of smoking on reducing body weight, support or advice on how to stop smoking from a family member or lecture on the harmful effects of smoking. Previous studies have reported gender differences in responses to smoking cessation messages [13] as well as tobacco cessation interventions [14,15].

We also assessed whether the beliefs about the effect of smoking on weight was associated with having quit smoking. In the GYTS survey adolescents had been asked whether they thought that smoking was associated with weight gain or weight loss. Adolescents who believed that smoking makes one's weight decrease were more likely to have stopped smoking among male adolescents, and less likely to have stopped smoking among female adolescents. What does this mean in the context of Ukraine? In many of the Western countries and lately in emerging economies, female adolescents in general perceive themselves to be overweight or heavy. Lean body weight is desirable. In many of these settings also, smoking is perceived as resulting in lean body weight [16-18]. Some female adolescents smoke in order to achieve or maintain a lean body weight [19,20]. Different perceptions of body weight may be different between genders and may explain why male adolescents were more likely to stop smoking in favour of a heavy body weight.

The finding that while male adolescents who received support or advice from family members were less likely to stop smoking, and female adolescents were more likely to stop smoking suggest that female adolescents took family members as credible source of information on cessation of smoking while male adolescents did not.

We found that having had a lecture on the harmful effects of smoking was associated with having quit smoking. A meta-analysis of adolescent smoking cessation programs reported by Sussman et al [21] suggested that programs that included a motivation enhancement component, cognitive-behavioural techniques, and social influence approaches were more likely to have been successful. These authors also reported that higher quit rates were found in school-based clinic and classroom modalities and in programs consisting of at least 5 quit sessions. This finding is consistent with our finding among female adolescents who were more likely to stop smoking after having had a lecture on harmful effects of smoking. Although we do not have a description of the content, conduct, frequency and number of lectures that had been delivered to adolescents who reported to have had a lecture, it is still heartening to note that having had a lecture on the harmful effects of smoking was associated with less likelihood of having to quit smoking among male adolescents.

### Limitations of the study

Due to the cross sectional nature of the study, it is not possible to confirm whether the factors that were identified as associated with having quit smoking preceded or followed quitting. The study also relied on self-reported history of having quit smoking. We did not verify that the adolescent had indeed quit smoking, for instance by using biomarkers such as exhaled carbon monoxide. We also did not have data on the nature of interventions that adolescents who reported having received help from a health professional obtained. Furthermore, it is unknown whether the adolescents who reported to have stopped smoking actually purposefully stopped the behaviour or just discontinued it.

## Conclusions

We have identified some factors that are associated with having quit smoking that are gender-specific. We suggest that adolescents' smoking cessation programs in Ukraine consider these factors in the design, implementation and evaluation of their gender sensitive programs guided by the FCTC that Ukraine ratified in 2006.

## Competing interests

The authors declare that they have no competing interests.

## Authors' contributions

AH conducted the analysis and took part in the interpretation of the results; SS conceived the analysis plan, reanalysed the data and participated in the interpretation of the results; ASM led the drafting of the manuscript; and ER participated in the interpretation of the results and writing of the manuscript. All authors read and approved the final draft for submission.
